# EMERITI PROFESSORS’ INTERGENERATIONAL TEAM TALK: REDUCING STUDENTS’ AGING ANXIETY ADDRESSES AFU PRINCIPLES

**DOI:** 10.1093/geroni/igad104.2075

**Published:** 2023-12-21

**Authors:** Ramraj Gautam, Karen Devereaux Melillo, Lisa Abdallah, Patcharee Wangwun

**Affiliations:** University of Massachusetts Lowell, Lowell, Massachusetts, United States; University of Massachusetts Lowell, Lowell, Massachusetts, United States; University of Massachusetts Lowell, Lowell, Massachusetts, United States; University of Massachusetts Lowell, Lowell, Massachusetts, United States

## Abstract

Intergenerational learning is one of the six pillars of the Age-Friendly University (AFU) institutional activity. This study was guided by the AFU principles 9 (involving university’s own retired community) and 4 (promote intergenerational learning between learners of all ages) and Positive Education about Aging and Contact Experience (PEACE) Model (Levy, 2018). A team of 4-5 students (12 teams total) in an undergraduate Introduction to Gerontology course in Fall 2022 conducted two, one-hour in-depth Zoom Intergenerational Team Talk (ITT) interviews with emeriti professors. The objective was to assess the change in students’ aging anxiety (measured using Bousfield and Hutchison’s, 2010, 4-item Aging Anxiety Scale), expectations about aging (measured using Sarkisian et al, 2005, 12-item Expectations Regarding Aging survey), and career and academic trajectories before and after the implementation of the ITT interview. The University IRB approved study sample included 57 students, where 31completed both pre and post surveys. A paired sample t-test showed a significant reduction in students’ aging anxiety [t(29) = 2.45, p = 0.020] and increased positive expectations regarding aging [t(26) = -2.94, p = 0.007], after the ITT interview activity. The results support the PEACE model that included emeriti professors as positive role models in an intergenerational contact. Emeriti were a valuable resource for lowering aging anxiety and increasing positive expectations regarding aging among university students. Engaging emeriti professors can create a more inclusive and age-friendly environment. Future studies should also explore the impact of intergenerational contacts on emeriti professors’ perception of younger adults.

